# Resilience and vulnerability of post-ostomy patients with early-onset colorectal cancer from the perspective of social-ecological theory: a qualitative study

**DOI:** 10.3389/fpsyt.2024.1497428

**Published:** 2025-01-21

**Authors:** Fangfang Yang, Fangming Feng, Hongming Gu, Han Liang, Jin Zhang, Yusha Cheng, Weiying Zhang

**Affiliations:** Department of Nursing, Shanghai East Hospital, School of Medicine, Tongji University, Shanghai, China

**Keywords:** resilience, social-ecological theory, post-ostomy, early-onset colorectal cancer, qualitative research

## Abstract

**Background:**

The incidence rate of colorectal cancer (CRC) is rising among adults under the age of 50 (early-onset colorectal cancer, EOCRC). Post-ostomy dysfunction, along with negative perceptions due to incorrect public views and a decline in quality of life, has a significant impact on these individuals, their families, and social relationships.

**Objective:**

By understanding the adaptation process of post-ostomy EOCRC(POEOCRC) patients, this study aims to provide information for developing targeted nursing interventions for this population.

**Methods:**

Based on the social-ecological theory, semi-structured, in-depth interviews were conducted with 16 POEOCRC patients in China between May 2023 and January 2024.Colaizzi’s method of phenomenology was employed for data analysis.

**Results:**

This study found two aspects of adaptation experience in POEOCRC patients. For resilience, three themes emerged:(1) Micro-Positive individual psychological experiences, (2) Meso-Positive adjustment within the family and (3) Macro-Social resource integration and utilization. In terms of vulnerability, three themes were as follows:(1) Micro-Persistent negative experiences, (2) Meso-Family crisis caused by the ostomy and (3) Macro-Urgency and fragility of social support.

**Conclusion:**

This study based on the social-ecological theory and highlights different dimensions of resilience and vulnerability experienced by POEOCRC patients. Early and targeted interventions to promote patients’ coping skills and their ability to adapt to family and society.

## Introduction

Colorectal cancer (CRC) has become a major global public health issue, ranking third in incidence rates and second in mortality rates. In 2022, there were an estimated 1.92 million new cases of CRC worldwide, with 592,232 cases reported in China alone, posing a significant threat to public health (1). Despite recent improvements in the availability of CRC screening and colonoscopies, it is worth noting that approximately 10% of all newly diagnosed CRC patients are diagnosed with early-onset CRC (EOCRC), defined as having an onset age below 50 years, and the incidence of this patient group has continued to increase over the past 10 years (2). Surgery is the main therapeutic modality for the treatment of CRC. In China, there are more than 1 million enterostomy patients, with over 100,000 new cases annually (3). The presence of an ostomy is a highly stressful event for EOCRC patients, as they play significant roles within their families and society due to their unique age characteristics. This major change of body structure, along with the resulting disruption to body image and diminished quality of life, has altered their physiological, psychological and social relationships (4–6).

Resilience is defined as the ability to adapt or rebound successfully after adversity, which is a crucial psychological trait for enhancing positive adaptation and managing negative emotions (7). Additionally, resilience is a positive outcome that can help individuals adapt to difficulties, maintain physical and mental well-being, and improve their health-related quality of life (8, 9). Resilience has been studied within various theoretical frameworks, including the Family Resilience Model (10), the Resilience in Illness Model (11), and the Resiliency Model of Stress, Adjustment, and Adaptation (12). The research results have demonstrated that resilience is influenced by the socio-ecological systems individuals rely on and operates as a dynamic, two-way process (13). This implies that examining patient resilience from a socio-ecological systems perspective is also warranted.

However, unlike deep understanding about the resilience in breast cancer (14) and lung cancer (15), there were limited research focusing on post-ostomy EOCRC(POEOCRC)patients. Quantitative studies reported that resilience in CRC patients was positively and directly associated with their quality of life (16), and could mediate the relationship between post-traumatic growth and perceived social support (17). To the best of our knowledge, no qualitative research has yet described the resilience of POEOCRC patients from a socio-ecological perspective. The resilience experienced by POEOCRC patients could be quite different from that of other cancer populations. The changes and impact of post-ostomy are manifested in more negative behaviors. Vulnerability is a characteristic of socio-ecological systems related to resilience, which significantly affects patients’ mental health and social support (18). Therefore, it’s necessary to emphasize the importance of regulating vulnerability to better adapt and promote resilience based on the social ecological theory.

The social-ecological theory emphasizes the interaction between individual development and the surrounding environment to form a complete ecosystem, which is divided into three basic types: microsystems, mesosystems, and macrosystems. Microsystems refer to individual systems, including biological, psychological, social, and other subsystems that influence individual behavior. Mesosystems refer to small-scale groups, including families, work groups, and other social groups. Macrosystems refer to systems larger than small-scale groups, including cultures, communities, institutions, and organizational structures (19, 20). The theory has been widely used in social work interventions, chronic disease management, and health promotion research (13, 21, 22), playing an important guiding role.

Therefore, this study aims to describe the adaptation process of POEOCRC patients within the context of Chinese culture. We hope that this study will inform the development of a targeted psychological care intervention program for this population.

## Methods

### Design

A qualitative study employing semi-structured interviews was conducted from a realist perspective (23), based on the social-ecological theory (19). This study strictly adhered to the Consolidated Criteria for Reporting Qualitative Research (COREQ) guidelines (24).

### Participants

Participants were recruited from a tertiary hospital in Shanghai, China, between May 2023 and January 2024. Participants were recruited using purposive sampling. The inclusion criteria for participation were as follows: (1) being pathologically diagnosed with EOCRC (CRC with onset <50 years) and having undergone permanent ostomy within the past year, (2) being over 18 years of age, and (3) being able to communicate in writing or speech. Exclusion criteria included: (1) having a palliative intent of treatment, (2) having severe mental illness or cognitive impairment, and (3) recovering during the perioperative period. The sample size of the study participants was determined by data saturation, with no new themes emerging as the standard for data collection. To maximize the sample variation, patients with different sociodemographic information were assessed.

### Data collection

Data were mainly collected via in-depth, face-to-face interviews. According to the social-ecological theory and the relevant literature, the research team employed a semi-structured interview guide. The group was composed of primary researchers, oncology specialist nurses, and psychologists. Two research subjects were selected for pre-interviews, and the final interview outline was revised and determined accordingly. Example questions included the following: How do you feel and what are your experiences after being diagnosed with CRC and undergoing an ostomy? How have your emotions and psychological state changed after the ostomy? What impacts and changes has the ostomy brought to your family, daily life, work, and social life? How do you actively cope with and adapt to these effects and changes in different situations? What support and assistance have you received during this period? What other areas would you like to receive help in? What are your expectations for the treatment and recovery of the disease and for the future?

We built trust with the participants, explained the purpose and significance of this study. After obtaining written informed consent, one-on-one interviews were held in quiet, private rooms. The interviews were recorded with audio and notes, capturing non-verbal cues like facial expressions and body movements. Any unclear information was confirmed with the interviewees. Each interview lasted 30-40 minutes.

### Data analysis

Within 24 hours after the interviews, the data were transcribed and checked verbatim by two researchers using recording software. The transcriptions were then proofread and supplemented with field notes. Subsequently, the data were extracted, coded, and analyzed using Colaizzi’s method of phenomenology (25):Carefully review the primary data; Analyze significant statements; Summarize and refine the meaning; Identify common characteristics or concepts to form themes, theme clusters, and categories; Connect the themes to the phenomena under study and describe them comprehensively; Recognize similar concepts and refine the themes; Return the findings to the interviewees for validation. Different points of view were discussed until a consensus was reached. Representative examples were extracted from the data as themes and sub-themes gradually formed.

### Ethical approval

The study received ethical approval from the Research Ethics Committee (Number:2023-032). All participants were provided with information about the study’s objectives, the data collection methods, the potential benefits and risks of involvement, the confidentiality measures in place, the exclusive use of data for research purposes, and their rights to withdraw from the study at any point.

## Results

Of 20 patients approached, 16 patients agreed to take part in the interview, while the other 4 patients did not complete the interview. Among them 3 patients withdrew from the study, and 1 patient abandoned treatment without undergoing surgery. Participants were coded as N1 to N16. Characteristics of participants are shown in **Table 1**. Slightly more males (n = 10) than females (n = 6) participated, with ages ranging from 27 to 49 (41.56 ± 7.56) years.

**Table 1:** Participant characteristics.

Variables	Number	Frequency (%)
Age (y)		
20-29	2	12.5
30-39	4	25
40-49	10	62.5
Gender		
Male	10	62.5
Female	6	37.5
Residence		
Rural	7	43.8
Urban	9	56.2
Employed		
Yes	11	68.8
No	5	31.2
Marital status		
Unmarried	2	12.5
Married	14	87.5
Religion		
Yes	2	12.5
No	14	87.5
Postoperative period(m)		
1-6	7	43.8
7-12	9	56.2
Stoma type		
Ileostomy	10	62.5
Colostomy	6	37.5
Stoma complications		
Yes	3	18.8
No	13	81.2

**Figure 1:**
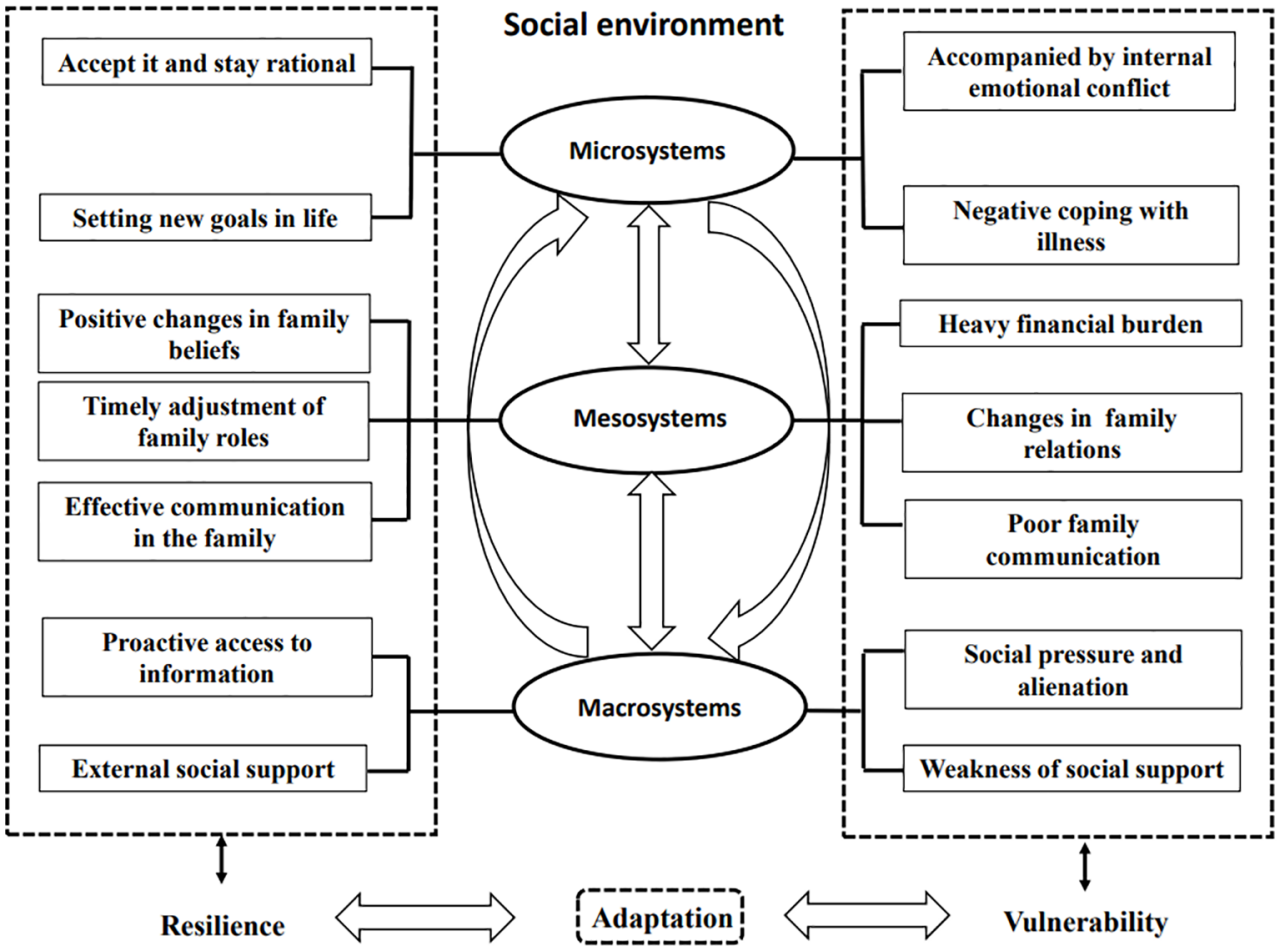
Resilience and vulnerability in POEOCRC patients.

The results of this study showed two different aspects of adaptation in POEOCRC patients. Some adapted well and demonstrated resilience, while others exhibited maladaptation and vulnerability. According to the social-ecological theory, themes were categorized separately for resilience and vulnerability. **Table 2** summarizes the refined themes of this study, and **Figure 1** illustrates these themes in the context of a logical and sequential model.

**Table 2:** Classification of theme and subtheme.

	Resilience		Vulnerability	
	Theme	Subtheme	Theme	Subtheme
**Micro-**	Positive individual psychological experiences	Accept it and stay rational	Persistent negative experiences	Accompanied by internal emotional conflict
		Setting new goals in life		Negative coping with illness
**Meso-**	Positive adjustment within the family	Positive changes in family beliefs	Family crisis caused by the ostomy	Heavy financial burden
		Timely adjustment of family roles		Changes in family relations
		Effective communication in the family		Poor family communication
**Macro-**	Social resource integration and utilization	Proactive access to information	Urgency and fragility of social support	Social pressure and alienation
		External social support		Weakness of social support

### Resilience

#### Theme 1 Micro-positive individual psychological experiences

##### Subtheme 1.1 Accept it and stay rational

As the post-ostomy period progressed, the interviews revealed that eight respondents were beginning to cope with setbacks and traumas and were accepting them openly. All of them were 7 to 12 months post-ostomy.

*Now it’s all about being positive and accepting it……. If you can’t get out from down there, you’ll have to get out from here. (N10)*

*I believe that anything is possible as long as the person is still alive, and I’m now slowly getting used to my stoma…… (N14)*

During the interviews, respondents with higher cognitive abilities shared their views on life, reflected on the meaning of living, and recounted their experiences and memories of the past.

*People come to this world as part of a journey. No matter what kind of burdens they carry, they have to experience the cold, taste the sweetness and sourness, and this is life! (N12)*

##### Subtheme 1.2 Setting new goals in life

Five respondents claimed that they tried to set new goals after their ostomy and treatments. By setting new life goals, they could gain a sense of control over disease and experience a renewed sense of hope.

*I plan to travel around with my stoma……Now science and technology are so advanced, I’m sure tomorrow will get better and better. (N5)*

*I used to be all about work, but now my goal is to spend time with my family and friends, and live each day to the fullest! (N11)*

#### Theme 2 Meso-positive adjustment within the family

##### Subtheme 2.1 Positive changes in family beliefs

Positive changes in family beliefs brought about a favorable rehabilitation environment and family atmosphere for six respondents. This led to more harmonious family relationships and increased their confidence in resuming normal life and recovering from illness as well.

*I was very beautiful when I was young, but now I’m so upset about my appearance. My husband told me to imagine my stoma as a rose, just like any other organ in my body, and not to reject it. (N7)*

*After I got this disease, I meticulously muddled through my life. My families are Buddhist, and now I’m Buddhist too. I’m thankful and full of strength every day. (N16)*

The positive attitude of families towards disease, together with the positive power of family beliefs, has a significant impact on improving the patients’ ability to cope with the disease.

##### Subtheme 2.2 Timely adjustment of family roles

Five respondents reported that they had to relinquish their family responsibilities after their ostomy. The roles and division of labor among other family members were adjusted accordingly.

*After I got sick, my in-laws moved in to help look after the kids. My father-in-law cooks all the meals, and I hardly do any housework now. (N5)*

*At first, my daughter(16years) changed my stoma bag. I was especially touched and relieved. At that moment, I felt that my daughter had grown up and taken the initiative to take care of her mother. (N9)*

##### Subtheme 2.3 Effective communication in the family

Six respondents said that opening up and discussing new living ways with their families raised their confidence and hope for the future.

*Since having a stoma, we often discuss together haw to eat better, how to exercise, and how to dress……We talk about anything to each other. (N2)*

*I talk to my husband right away if I need anything, and he doesn’t mind at all. We communicate about any problems we have. (N8)*

#### Theme 3 Macro-social resource integration and utilization

##### Subtheme 3.1 Proactive access to information

Eight respondents indicated that they would proactively seek disease-related knowledge and rehabilitation information through various channels such as the Internet and multimedia sources. Proactive information-seeking support enables them to solve better.

*Now, all the information about knowledge of diseases, treatments, and medical experts can be looked up on the Internet. (N12)*

*I have joined many groups and followed a lot of public accounts related to stoma care. I have watched numerous videos related to situations similar to mine and those teaching how to care for my stoma. (N1)*

##### Subtheme 3.2 External social support

All respondents pointed out that various forms of social support play a crucial role in the disease recovery. For example, calls from relatives and friends, even just to say hello, and financial support provided great comfort to the patients.

*Relatives and friends have been calling me, saying that if there were any financial difficulties, I could just need to let them know. (N3)*

The professional care from the medical staff and the interaction among patients greatly alleviated the respondents’ anxiety about their illness.

*At first, we were not very skilled in changing the ostomy bag. The nurse was especially patient and guided us well. I think our nurses here are really good and very responsible. (N13)*

*Usually, I also communicate with my fellow patients about their conditions, and knowing that they are getting better has increased my confidence. (N10)*

In addition, the health insurance policy also alleviated part of their financial pressure.

*Now that the health insurance also reimburses part of it, the financial pressure is not as great. (N11)*

### Vulnerability

#### Theme 4 Micro-persistent negative experiences

##### Subtheme 4.1 Accompanied by internal emotional conflict

Almost all the POEOCRC patients had a physical condition and found it difficult to accept for a while. As the physical damage caused by ostomy couldn’t be recovered in a short period of time, patients were likely to suffer from impatience, anxiety, and other psychological problems.

*For a while, I was grumpy whenever I saw the stoma on my body. I often lost my temper with my family for no reason and found it difficult to cope with everything at once. (N1)*

Half of the respondents indicated that shame and low self-esteem had also arisen, creating a stigma.

*After undergoing a stoma, stool would often come out. It smells bad and I think it’s dirty. (N4)*

*At first, I couldn’t take it, I just felt humiliated…… (N17)*

##### Subtheme 4.2 Negative coping with illness

There were three respondents who resorted to negative coping, such as losing interest in everything, delaying medical treatment, or even relieving pain by relying on medication.

*I’m like a zombie now. I can’t be interested in anything, and I don’t want to do anything. (N2)*

*At first, my stoma skin was a little red and itchy. I thought my hygiene habits were not good enough, so I didn’t go to the stoma clinic because I thought it would get better on its own. (N8)*

*I’m now dependent on painkillers and sleeping pills for sleep well at night……(N12)*

#### Theme 5 Meso-family crisis caused by enterostomies

##### Subtheme 5.1 Heavy financial burden

Almost all respondents faced the possibility of disease recurrence after the surgery. They had to endure a lengthy, complex treatment process while enduring the financial burden of high medical costs.

*I’ve spent a lot of money on treating this disease, and I still need more treatment, so my family is really experiencing financial difficulties. (N10)*

*My family isn’t well off, and now that I’m sick, how can I not be under a lot of financial pressure? (N7)*

##### Subtheme 5.2 Changes in family relations

In family relationships, younger adult patients played multiple roles as children, parents, wives or husbands. They assumed the responsibilities of caring for their parents and teaching their children, while also being the main source of income for the family. Five respondents stated that they were unable to fulfill their corresponding responsibilities when they fell ill, which led to changes in family relationships.

*I can’t help my wife with the household chores. She has to go out to work for money and take care of the children at home. I can’t fulfill my responsibilities as a husband and a father……(N11)*

*How am I supposed to take care of my family when I can’t even do my regular chores (ugh)? (N10)*

##### Subtheme 5.3 Poor family communication

Most respondents in this study were middle-aged. Under the influence of our society and culture, the expression of emotional needs among family members is more subtle and introverted. Four respondents reported that they became silent and reduced communication with their families post-ostomy. Additionally, family members tried to avoid mentioning anything related to their illness. Moreover, over-protection between patients and their families led to a lack of family communication.

*I feel like there’s a haze over the family. My wife doesn’t talk much, the kids have distanced themselves from me, and there’s no laughter in the house. (N17)*

*My wife hid it from me at first for fear of upsetting me, only saying that it was a polyp and that it would be fine if she had it removed……When I found out the truth, I felt like the sky was falling, and I lost my appetite and stopped talking. (N13)*

#### Theme 6 Macro-urgency and fragility of social support

##### Subtheme 6.1 Social pressure and alienation

Almost all respondents felt the pressure from society due to changes in body shape and comfort level post-ostomy, as well as the public’s lack of understanding.

*With a stoma, people around you will look at you differently, and it’s very stressful. (N17)*

Four respondents started avoiding social activities because they feared that if their ostomy bags leaked, they would be stared at or even be seen as a bother.

*I’d rather stay home than go to public places, especially it’s hot now. And I’m afraid my scent will be smelled. If I do join others for a meal, I will avoid eating raw, cold, oily, or spicy food. (N3)*

*I usually participate in a lot of activities……but in the past six months I have not tried any activities, it is always inconvenient with this stoma. (N10)*

##### Subtheme 6.2 Weakness of social support

Two respondents who were in the workforce said they had experienced discrimination during the employment process.

*I can’t go out and find a job. Once the employing unit knows that you have a stoma, they will definitely dismiss you because they would have to bear the losses if you were injured during the work. (N8)*

Respondents spent most of their time at home after being discharged from hospital. Three respondents with a shorter postoperative period expressed a desire for more professional support and help with home care.

*I have an ileostomy. It’s all loose stools, and it’s not easy to care for. I have dermatitis rashes and little red bumps, and I don’t know what to do at home to make it better. (N9)*

## Discussion

This study described resilience and vulnerability in POEOCRC patients within the past year from the perspective of social-ecological theory. Digging deeper into the POEOCRC’s real experience of facing and coping with this disease under a specific cultural background.

The interactions within the socio-ecological systems of POEOCRC patients had an impact on the patients’ physical and mental health, as well as their ability of family and social adjustment. We should learn from their resilience, while as to their vulnerability, early targeted interventions are necessary for maintaining the stability of their socio-ecological systems.

We found that as the postoperative period following enterostomy progressed, especially around an average postoperative time of 7-12 months, individuals were able to face and openly accept the negative events they had experienced. At this stage they also became more thankful and began to cherish the present moment. Some patients with higher cognitive function started to reshape the meaning of their lives, establish new life goals, and enhance their self-perception, consistent with Luo (9) and Chou’s (26) study on resilience in colorectal cancer stoma patients. However, patients exhibited varying degrees of emotional internalization and negative coping during the treatment and recovery process, which is similar to the findings by Yi (27) and Qaderi (28) et al. Research has indicated that when people experience negative stressful events, both positive and negative mentalities coexist. When they cope with these events, a series of positive changes could occur including improving psychological adjustment, establishing closer connections with others, and enhancing self-perception (29). Therefore, healthcare professionals should pay attention to the positive changes in patients’ resilience during the process after CRC stoma surgery, which can help alleviate their negative emotions and promote positive coping. Meanwhile, by emphasizing the assessment of individual factors, timely detection of the patients’ psychological distress and treatment needs and promoting their positive adjustment abilities, implementing interventions aimed at enhancing patients’ psychological resilience can better improve their adaptation to the disease.

With the advancement of positive psychology, its approaches and practices not only promote well-being but also help to reduce mental illness, maintain mental health, and strengthen one’s psychological resources and capacities (30). The concept of family resilience has now shifted from focusing on problems to highlighting families’ positive responses to adversity. This shift emphasizes the use of family power and resources for the patients’ adaptation (31, 32), which provides a new perspective for cancer patients’ family-centred care interventions. Walsh argues that family resilience is built through interactions among individuals, families, and the external environment, summarizing three key dimensions: family belief systems, organizational patterns, and communication processes (10). This study, focusing on POEOCRC patients at the meso-level, found that positive family coping aligns more closely with the theory of family strengths, consistent with studies by Zhu (33) and Yang (34) et al. Improved 5-year survival rates for CRC patients means longer time spent with family members (35). Through positive changes in family beliefs, timely adjustments of roles, and openness to new life experiences, families could better cope with the crises and challenges brought by enterostomy. Previous studies have only explored the individual resilience perspective (26, 36). However, in order to achieve better adjustment and adaptation, family resilience has an indispensable impact on the recovery process of the patient (37–39). Therefore, healthcare professionals should provide targeted interventions from the perspective of family strengths to promote positive mindsets and coping strategies. It is recommended to apply the theory of family resilience (10, 40) to guide clinical practice, enhance the training of medical staff, comprehensively assess family resources for POEOCRC patients, and conduct relevant thematic courses and psychotechnical skills training through family group meetings (41, 42). This approach aims to formulate a diversified program of intervention and practice for family resilience.

Previous studies have shown that social support is generally beneficial for patients by enhancing their resilience and adaptability (36, 43).Our study, focusing on POEOCRC patients at the macro-level, also reflects this condition. However, research has showed lower psychosocial resilience in patients with less than 1 year post-ostomy (44). This study also found that POEOCRC patients were under greater social pressure as well as experiencing social alienation, which is consistent with the findings of Song et al (45). In some rural areas, suffering from cancer is considered as having done something bad in a previous life. They also inevitably face the finger-pointing criticism from their neighbors in the village due to the odor from stoma. In turn, patients with POEOCRC tend to conceal their inner feelings with silence. Furthermore, the concept of “patience” in traditional Chinese culture influences the patients who are more willing to bear the pain alone and thus become more socially isolated (46).Therefore, society should be more aware of this disease. Healthcare professionals should focus on POEOCRC patients’ self-expression and apply psychological interventions such as supportive expression group therapy to guide self-psychological regulation, which can help improve emotional expression and foster social interactions.

Furthermore, in this study, the majority of respondents highlighted the importance of care continuity, home professional support, national healthcare protection, and supportive policies. To enhance patients’ social adaptation levels, it is crucial to effectively integrate external social resources, providing multi-channel and multi-dimensional social support. For instance, the ‘hospital-family-community’ tripartite care model promotes care continuity, reduces home care burdens, and improves quality of life. Based on the concept of shared decision-making (47), a family-centered collaborative care model has been developed to encourage family members’ participation in the patients’ care planning as well as treatment decisions. Peer support groups, dynamic two-way feedback via online platforms, and regular knowledge sharing on disease care and problem-solving are all very essential. Additionally, we advocate for society to treat these patients as ordinary person, reducing excessive sympathy and concern and giving sufficient respect in the meantime. Policy reforms are needed to prevent employment discrimination for ensuring the normal employment for capable younger adult patients. For families facing financial challenges, adjusting and supplementing to national healthcare insurance can address these challenges.

## Limitations

There were some limitations in this study. Firstly, the sample questions focused more on the physical and psychological consequences of post-ostomy and patients’ coping strategies. This narrow focus may affect the comprehensiveness of the study’s results and could benefit from expanding the sample in future research. Secondly, the sample selection was limited in space, time and population. The data in this study were cross-sectional, which may not accurately capture the evolving experiences of resilience and vulnerability of POEOCRC patients over time. Additionally, interviews were conducted in a single hospital in China, and the views of the participants were influenced by the medical environment. Furthermore, only patients were interviewed, resulting in a rather homogenous perspective. Future research should consider using a longitudinal design, conducting community-and home-based interviews, and including both caregivers and other family members to provide a more diverse perspective. Finally, the study was grounded in social-ecological theory but lacked a clear theoretical framework addressing resilience and vulnerability. We should integrate relevant theoretical frameworks together as the research basis in future research.

## Conclusion

This study provided an in-depth insight into the resilience and vulnerability of 16 POEOCRC patients from the perspective of social-ecological theory. Combining specific cultural backgrounds, multidisciplinary and personalized nursing interventions should be developed from the micro, meso, and macro systems of POEOCRC patients, encompassing individual, family, and societal dimensions, to improve patients’ resilience and adaptability.

## Data Availability

The raw data supporting the conclusions of this article will be made available by the authors, without undue reservation.
